# Automated micro-solid-phase extraction clean-up and gas chromatography-tandem mass spectrometry analysis of pesticides in foods extracted with ethyl acetate

**DOI:** 10.1007/s00216-023-05027-5

**Published:** 2023-11-16

**Authors:** Andreas Schürmann, Claudio Crüzer, Veronika Duss, Robin Kämpf, Thomi Preiswerk, Hans-Joachim Huebschmann

**Affiliations:** 1 Cantonal Laboratory Zürich, Official Food Control Authority of the Canton of Zürich, Department Pesticide Analysis, Zurich, Switzerland; 2 CTC Analytics AG, Zwingen, Switzerland

**Keywords:** Pesticide residues, Ethyl acetate extraction, Automated sample preparation, Micro-SPE extract clean-up, GC-MS/MS

## Abstract

Generic extraction methods for the multi-compound pesticide analysis of food have found their solid place in laboratories. Ethyl acetate and acetonitrile extraction methods have been developed as fast and easy to handle standard multi-compound methods, both feature benefits and limitations. The direct injection to gas chromatography can be impaired by a high burden of coextracted matrix, resulting in deterioration of the chromatographic system and matrix effects, requiring frequent maintenance. Therefore, common clean-up methods, such as dispersive solid-phase extraction, freeze-out of fats, or gel permeation chromatography, have been applied in clean-up. Automated clean-up using micro-solid-phase extraction (µSPE) is a recent development with several demonstrated advantages when employed in the analysis of pesticides and other contaminants in foods extracted with acetonitrile, but it has not yet been evaluated in this application using ethyl acetate for extraction. In this study, an automated procedure using µSPE cartridges was developed and established on an x,y,z robotic sampler for the raw extract clean-up and preparation of diluted samples for injection on a GC-MS/MS system. Validation experiments for 212 pesticides, polychlorinated biphenyls, and polycyclic aromatic hydrocarbons in lettuce, avocado, raspberry, paprika, egg, and liver extracts were performed using µSPE with MgSO_4_, PSA, C_18_, and CarbonX. The performance in routine operation is briefly discussed.

**Graphical abstract:**

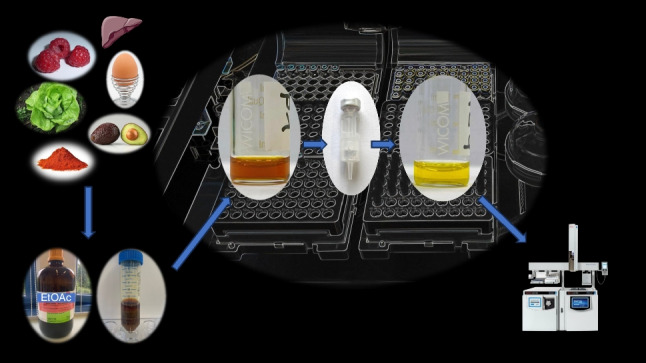

**Supplementary Information:**

The online version contains supplementary material available at 10.1007/s00216-023-05027-5.

## Introduction

The rising number of samples and analytes plus the demand for swift processing and reporting for multiresidue and multimatrix pesticide analysis in routine food control requires a generic uniform and capable extraction step, and most desired a consistent clean-up procedure. These requirements call for a reliable automation to replace the time-consuming manual sample preparation, in particular allocated in the traditional multi-step extract clean-up using sorbent material mixes, or the traditional fat removal for different kinds of food commodities, especially from high-lipid-containing foods, before GC-MS/MS analysis.

Currently, the prevailing sample preparation approaches for routine pesticide analysis are different versions of the quick, easy, cheap, effective, rugged, and safe (QuEChERS) method using acetonitrile (MeCN) for extraction [1]. Ethyl acetate (EtOAc) has a long history as an extraction solvent [2–6]. In the meantime, EtOAc is used for extraction only by approximately 7% of monitoring labs, such as those that follow the Swedish EtOAc (SweEt) method. The latter is in use since 1989 in the monitoring of pesticide residues also for the more polar pesticides in fruit, vegetables, cereals, and samples of animal origin with high recoveries [7]. The EtOAC method is used for a wide range of pesticides, having many advantages compared to the MeCN approach. EtOAc is especially suitable for the extraction of high-sugar commodities since sugar has limited solubility in EtOAc [8]. It is also reported that compounds such as captan, captafol, folpet, endrin, and iprodione yield high recoveries [9, 10] due to their instabilities in MeCN. In addition, there is a more than two-fold cost advantage and less toxicological concern with the use of EtOAc compared to MeCN. In some cases, there is a down-side, as EtOAc also extracts a large amount of non-polar co-extractives, such as lipids and wax materials, which must be removed in extracts of fatty matrix samples before the chromatographic determination. The Official Food Control Authority of the Cantonal Laboratory in Zürich chose the EtOAc extraction method (acetate-buffered) combined with an optional clean-up step for gas chromatography of fat-containing extracts; in the past by gel permeation chromatography (GPC) [11], in the meantime by freeze-out or dilution*.*

Using the suggested dispersive solid-phase extraction (dSPE), it turned out in practice that different food commodities required different adequately modified sorbent mixes to optimally handle the many diverse matrix components like chlorophyl, carbohydrates, or lipids in the extracts without sacrificing the recovery of the large set of target pesticides. The automation of the clean-up procedure using micro-SPE cartridges (µSPE) was first investigated by Morris et al. (2014) by replacing the manual dispersive SPE with miniaturized SPE cartridges for the clean-up of QuEChERS MeCN extracts from fruits, spices, and concentrated food ingredients [12]. An automated processing was established that uses commercially available x,y,z robotic samplers and compatible miniaturized SPE cartridges. A workflow program controls the robot for the cartridge conditioning, loading of the raw extract onto a cartridge, and elution of the cleaned extract into regular autosampler vials for an optional dilution, addition of analyte protectants or standards, and finally the injection for analysis.

Extensive development work was invested in the validation of different clean-up sorbent material mixes for use with GC-MS and LC-MS analysis for fatty matrices [13, 14]. The optimized sorbent mix in the µSPE cartridges for GC-MS analysis was decided to contain 45 mg of a mixture of PSA, C_18_, CarbonX, and MgSO_4_ materials. Using MeCN as the extraction solvent, Lehotay et al. (2016) used the same type of µSPE cartridges with a slightly modified workflow for a fast extract clean-up from vegetables, fruits, and even high-fat-containing fish samples, in-time with the chromatographic runtime [15].

A large number of automated applications using µSPE for extract clean-up [16] have been published since for pesticides from different critical [14, 17] and fatty matrices [18], environmental contaminants [19, 20], veterinary drugs [21], polycyclic aromatic hydrocarbons in food oils [22], or even drugs in biological materials [23, 24]. While these µSPE applications typically use MeCN as the extraction solvent, the described procedure to our knowledge for the first time uses EtOAc as the extraction solvent and automated µSPE clean-up of the raw extracts of food.

This new technique must be further optimized as gas chromatographic performance can be lost despite the µSPE clean-up when repeatedly injecting EtOAc extracts of complex matrix samples, such as spices or very fatty samples like avocado. It is shown that a few such lipidic matrix samples can be run in a routine sample series including other fruits and vegetables with less matrix effects without drastic effects on chromatography. Due to the different extraction properties of EtOAc compared to MeCN, e.g., more lipids and less sugars extracted, the chromatography and detection of pesticide residues can be influenced dissimilarly. Validation experiments for a large set of pesticides, polychlorinated biphenyls, and polycyclic aromatic hydrocarbons in lettuce, avocado, raspberry, paprika spice, egg, and liver extracts are presented.

## Materials and methods

### Standards and reagents

#### Pesticide standards, stock solutions, and solvents

The wide range of pesticide reference substances and a few other compounds (PCBs and PAHs) as listed in Table S1 (212 compounds and triphenyl phosphate (TPP) as procedural standard) were of high purity and bought from Sigma-Aldrich (Buchs, Switzerland), HPC Standards (Borsdorf, Germany), and Ehrenstorfer (Augsburg, Germany). A mix solution also containing PCBs was purchased from Ehrenstorfer (Mix 13 with PCB congeners 28, 52, 101, 153, and 180). All other stock dilutions were prepared with a concentration of 1 g/L in screw-capped bottles using the Quantos liquid dosing system (Mettler Toledo, Switzerland) and further diluted to obtain the working standard solutions (10 mg/L). All external standards contained cucumber matrix extract as an analyte protectant [25] (1 g/mL in EtOAc). EtOAc, p.a., was from Roth, water, HPLC grade, and acetic acid, p.a., from Scharlau and MeCN, HPLC grade, from Reuss Chemie in Switzerland.

#### Micro-SPE (µSPE) cartridges

Two different types of µSPE cartridges were used in this workflow. Most of the work was done using the cartridges supplied by Instrument Top Sample Preparation Inc. (ITSP, GA, USA). For the more challenging lipidic matrix paprika spice also the new PAL System µSPE cartridge design with a customized sorbent mixture was tested (CTC Analytics, Zwingen, Switzerland). The sorbent mix composition of the two cartridges is specified in Table 1. The described ITSP routine cartridge type and sorbent mix are used in common for all analyzed food matrices including high-fat-containing food. The cartridges are used once for each sample as due to the scavenging operation principle the extracted sample matrix is kept on the cartridge while the cleaned extract is collected for subsequent GC-MS analysis [26].

**Table 1:** µSPE cartridge sorbent material composition

	ITSP routine cartridge		PAL System complex matrix cartridge	
Sorbent	Bed mass (mg)	%	Bed mass (mg)	%
PSA	12	27	8.18	18
C18	12	27	8.18	18
CarbonX	1	2		
GCB			4.09	9
MgSO_4_	20	44	24.55	55
Total	45	100	45	100

#### Materials

Employed 50-mL polypropylene tubes were from Greiner, and 0.7-mL polypropylene autosampler vials, 2-mL clear glass autosampler vials, and 11-mm aluminium crimp caps with butyl/PTFE septum were from Wicom. QuEChERS sample preparation kits comprising 50-mL tubes with 6 g MgSO_4_ and 1.5 g sodium acetate and the DisQuE pouches for 50 mL CEN (1 g NaCitrate, 1 g NaCl, 4 g MgSO_4_) were from Waters.

### Instrumentation and analysis

All analyses were carried out on a Thermo GC-MS/MS System (Thermo Fisher Scientific, Austin, TX, USA) consisting of a TSQ 9610 triple quad with an advanced electron ion (AEI) source and a Trace 1310 GC equipped with a TriPlus RSH SMART robotic system (Thermo Fisher Scientific, Segrate, Italy) for automated µSPE extract clean-up and injection. The Xcalibur software (Thermo Fisher Scientific) was used for the execution of the sequence table with the automated sample preparation workflow, the GC-MS/MS instrument control, and data acquisition. Data processing and reporting was done using TraceFinder software version 5.1 (Thermo Fisher Scientific).

The GC was equipped with a temperature programmable injector (PTV) allowing the injection at a low initial temperature of 55 °C. Excess solvent vapor from a 3-µL injection volume is vented by applying a short-time split flow of 30 mL/min for 6 s, followed by the 3-min splitless completion of the vaporization and transfer of the analytes to the GC column. The PTV injector temperature was then ramped to 330 °C with 2.5 °C/s and kept for 12 min at this temperature. A standard baffled inlet liner without glass wool (Restek Corporation, Bellefonte, PA, USA) was used. For separation, a DB-5 ms Ultra Inert GC column (Agilent Technologies Inc.) was employed with 15-m length, 0.25-mm ID, and 0.25-µm film thickness.

Carrier gas used was helium (6.0 quality) in constant pressure mode at 70 kPa (depending on column length). The GC oven temperature program started at 55 °C (2 min), ramped to 165 °C with 20 °C/min, then to 205 °C with 3 °C/min, and finally to 310 °C with 10 °C/min (3 min). The total analysis time is 34.3 min, followed by a cool-down of approx. 6 min. The transfer line temperature to the MS was set to 290 °C constant. The mass spectrometer was operated in electron impact (EI) mode at an ion source temperature of 220 °C. The acquisition scan mode used was the timed SRM with a total of 425 MS/MS transitions for 213 compounds (2 transitions for all analytes and 1 transition for TPP).

### Sample preparation

#### Samples and homogenization

Six food samples representing diverse commodity groups were chosen. Samples of organic iceberg lettuce, avocado, raspberry, ground paprika spice, whole egg, and liver from enforcement sampling were analyzed preliminarily to confirm no detectable residues were present. All samples, except the paprika, were cryogenically homogenized with liquid nitrogen and a lab mill (Robot-Coupe R 5 V.V., Rotor Lips, Uetendorf, Switzerland) to achieve representative test portions [27, 28]. The homogenates were stored at − 30 °C.

#### EtOAc extraction

For lettuce, raspberry, and avocado, 10-g test portions of cryomilled sample were used, for egg 5 g, and for paprika spice and liver 2 g each. The paprika spice powder was soaked with 10 mL of water prior to extraction. Sample portions were transferred into 50-mL tubes with 6 g MgSO_4_ and 1.5 g sodium acetate (NaAc). Ten milliliters of EtOAc containing 1% acetic acid (HOAc) and the procedural standard (TPP) was added. The samples were extracted for 5 min on a mechanical shaker at 1000 rpm (Collomix VIBA X.30 V, Gaimersheim, Germany). After shaking, the samples were centrifuged (Vaudaux-Eppendorf 5810 R) at 3900 rpm for at least 2 min. The supernatant was used as raw extract for the subsequent automated µSPE clean-up. One milliliter of the raw extract was transferred to 2-mL autosampler vials and placed into the µSPE trayholder of the robot.

#### MeCN extraction

For avocado, also raw MeCN extracts of 5-g cryomilled samples were prepared according to the modified QuEChERS method published by the European Reference Laboratory for Single Residue Methods [29], but without the freeze-out step.

### Automated clean-up procedure and workflow

**Fig. 1:**
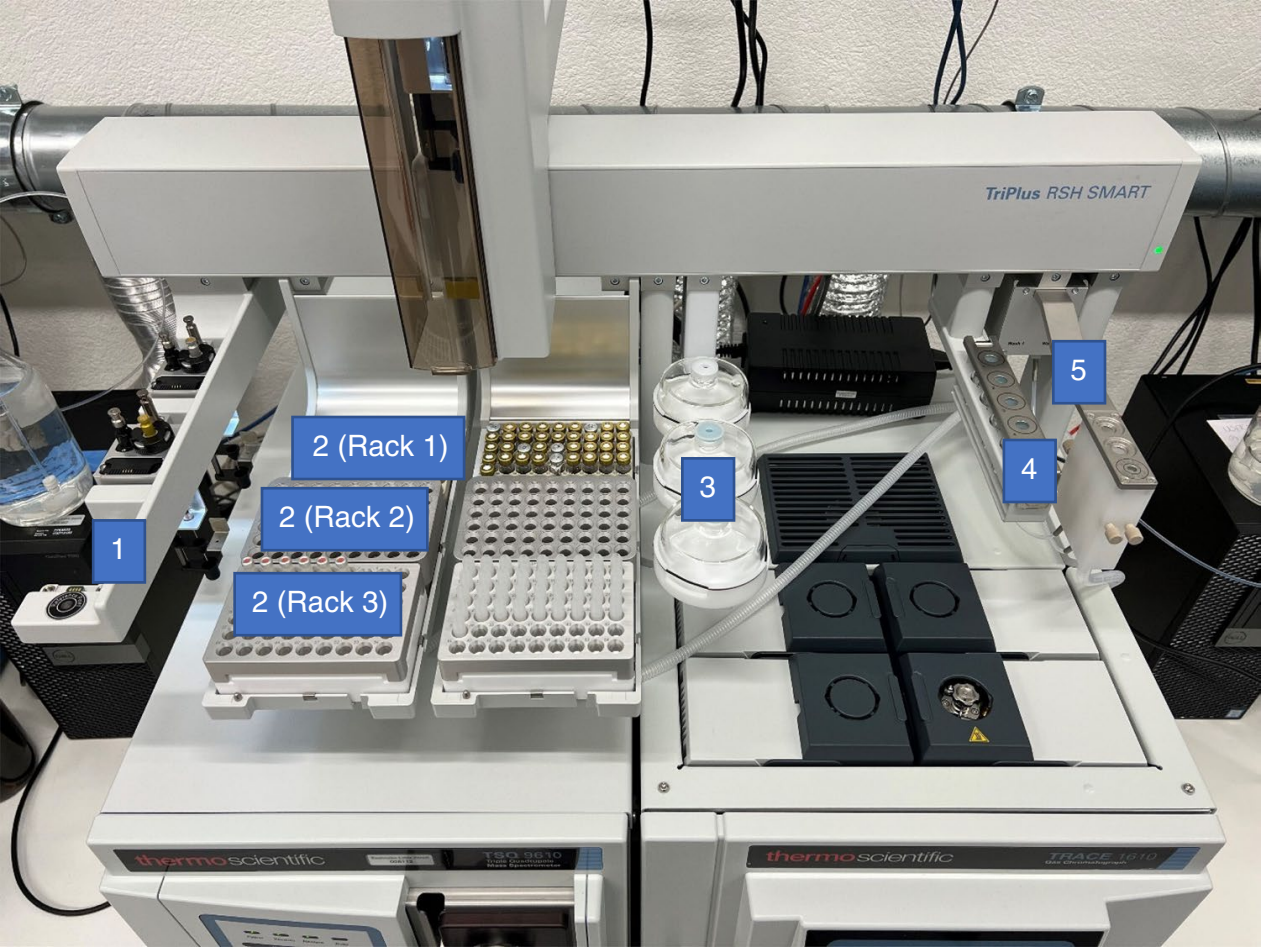
TriPlus RSH SMART system configuration for automated µSPE extract clean-up. 1, automatic tool change (ATC) station with 10-, 25-, and 1000-µL syringes; 2, µSPE tray with rack for raw extracts in 2-mL vials, elution rack into 2-mL vials, and µSPE cartridge rack; 3, solvent module for 3 × 100 mL reservoirs for solvents; 4, standard wash station for location of standards and APIs in 2-mL vials; 5, fast wash station for syringe cleaning

The configuration of the TriPlus RSH robotic system with the dedicated µSPE tray holder is shown in Fig. 1. The vials with the EtOAc or MeCN raw extracts and standards are placed into rack 1 of the µSPE tray holder. The eluted and cleaned extracts are collected in empty 2-mL vials in rack 2 in the center of the tray holder. Rack 3 in the front of the tray holder holds the rack with the µSPE cartridges for the clean-up workflow. The processing of the sample is executed serially including the injection of the cleaned extract to the GC-MS/MS.

The sample raw extracts and standards get processed with the ongoing GC separation of a previous analysis on a self-controlled time axis of the TriPlus RSH robot so that the cleaned sample is ready for injection when the GC ready signal is expected (“prep-ahead” mode). The samples get injected right after the clean-up step. Waiting times, in particular different waiting times after contact with the sorbent material, are avoided so that all samples are treated on the identical timeline to avoid uncontrolled decomposition thus improving reproducibility of the recovery of the target analytes.

**Fig. 2:**
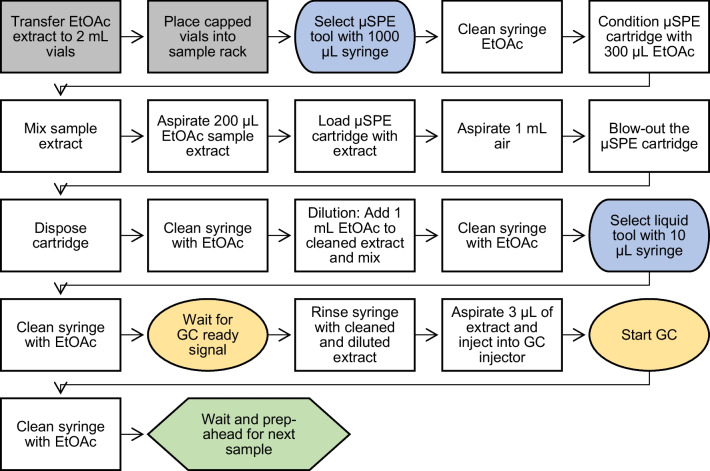
The automated µSPE clean-up workflow of an EtOAc raw extract with step-by-step activities (in grey the manual vial load onto the robot)

The workflow for the µSPE clean-up and GC injection procedure as illustrated in Fig. 2 was created in-house using the TriPlus RSH Sampling Workflow Editor software (Thermo Fisher Scientific, Segrate, Italy). The required tasks for the intended workflow were customized by adaptation of the default parameters. The sequence of activities is saved as the final clean-up workflow and executed from the sample sequence table of the TraceFinder data system.

#### Automated clean-up workflow

The automated clean-up workflow for all raw extracts and standards starts with the conditioning of the µSPE cartridges held ready in rack 3 with 300-µL elution solvent (EtOAc) from the solvent reservoir. Bypassing the conditioning step of the cartridges resulted in less reproducibility and thus higher overall deviations of results (data not shown). The pre-wash of the cartridges also helps reduce reagent background components that might interfere [30].

After the cartridge conditioning, the large volume preparation syringe loads 200 µL of the raw extract or standard from a sample vial in rack 1 and moves to the cartridge tray to pick a conditioned cartridge by inserting the needle. The cartridge is moved by the syringe to the elution tray and inserted into an empty vial held ready below the cover at rack 2 (Fig. 1). The sample is then pushed through the sorbent bed of the cartridge with a constant speed of 2 µL/s by the syringe. The extracted sample matrix is retained on the cartridge, the cleaned sample elutes and gets collected in the empty vial below. Additionally, a blow-out step using the empty syringe was added (1000 µL air). The fully detailed automated µSPE clean-up workflow is graphically illustrated in Fig. 2. After the clean-up procedure, the preparation syringe is cleaned with EtOAc and a three-stroke full volume cleaning step at the fast wash station.

#### Injection

Then the cleaned sample is diluted with 1 mL EtOAc using the 1000-µL syringe and mixed with five full volume strokes. This results in an approximately six-fold dilution for all raw extracts and standards. After the mixing step and a syringe cleaning step, the robot changes to the liquid tool with a 10-µL GC injection syringe and 3 µL of the final sample is injected using the normal injection mode. The injection syringe is cleaned after each injection with EtOAc with a three-stroke full volume cleaning step.

### Method validation

Recoveries were calculated using external standards in solvent with analyte protectant as described in the “Standards and reagents” section and standard spikes to the different blank matrices before extraction. Concentration ranges were from 0.001 to 0.1 mg/kg. Five injections of each extracted fortified matrix with the concentration levels 0.01 and 0.1 mg/kg, respectively, 3 injections with the levels 0.001, 0.005, and 0.05 mg/kg of the 212 compounds, were performed using the automated workflow as described. From these repeated measurements, recoveries and repeatability as relative standard deviations at each spiking level were calculated. Limits of detection (LODs) refer to the lowest spiking level with acceptable signal to noise ratio.

For each matrix, the following sequence of standards and sample extracts with 38 injections (all cleaned-up by µSPE) were run: hexane; standards 0.001, 0.005, 0.01, 0.05, and 0.1 mg/L; hexane; blank (a water sample processed instead of homogenate); unspiked sample; 11 spiked samples (3 × 0.001, 3 × 0.005, 5 × 0.01 mg/kg); hexane; standard 0.1 mg/L; hexane; 8 spiked samples (3 × 0.05, 5 × 0.1 mg/kg), hexane, standards 0.001, 0.005, 0.01, 0.05, and 0.1 mg/L; hexane. The scope of the method was later extended to the matrix liver. The applicability of the method for liver was demonstrated with 0.01 and 0.1 mg/kg spikes only (injected 5 times).

The effectiveness of the µSPE clean-up was estimated in all matrices by comparing residual solids in the extracts gravimetrically after evaporating the extraction solvent with a gentle stream of nitrogen.

## Results and discussion

### Overall results and optimization for different matrices

The initial validation concept to extract all samples only with EtOAc had to be abandoned. Repeated measurements during the validation sequence of avocado extracted with EtOAc led to continuous degradation of the gas chromatographic system after several injections. It was thus decided to extract avocado with MeCN (without a freeze-out step) to lower the fat burden in the raw extracts (compared to the EtOAc extracts) for the validation experiments. All other matrices were extracted with EtOAc without exception.

#### Avocado, EtOAc extraction

Avocado EtOAc extracts run in series during the validation measurements (without a freeze-out) step led to degradation of the chromatographic system after several measurements, visible as analyte-specific interferences such as severe retention time shifts and peak shape deterioration. In Fig. 6, the performance of TPP analysis is shown. Although the retention time begins to shift with each further injection of avocado extract due to the co-extracted lipids, there was no significant change of response.

#### Avocado, MeCN extraction

**Fig. 3:**
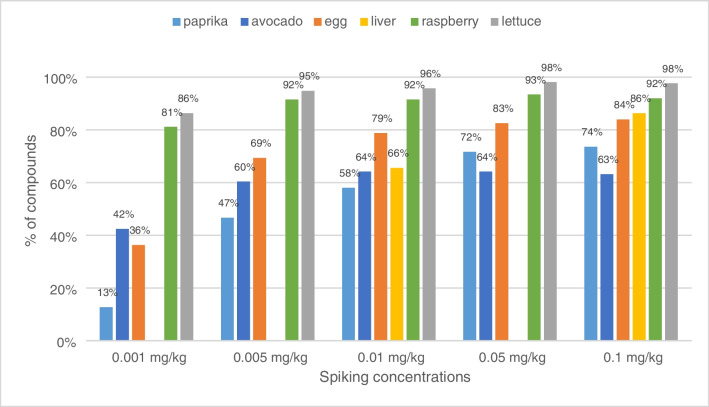
Percentage of the 212 compounds with recoveries within the range 70–120% and RSD ≤ 20% after clean-up of raw extracts with ITSP cartridges of spiked samples at five concentration levels: 0.001 (*n* = 3), 0.005 (*n* = 3), 0.01 (*n* = 5), 0.05 (*n* = 3), and 0.1 mg/kg (*n* = 5; liver only 0.01 and 0.1 mg/kg)

With the MeCN (without freeze-out), less lipids are co-extracted compared to the EtOAc extraction. The highest residual matter was determined for EtOAc raw extracts of avocado after clean-up (Table 2). This corresponds with the observed loss of the gas chromatographic performance after several injections. The low matrix burden of the sample extracted with MeCN is notable and displays the adequate clean-up for avocado, making tedious preceding freeze-out of fats in the raw extracts unnecessary. No retention time shifts were observed. A total of 63% (134 compounds) of the analytes passed the validation criteria at 0.1 mg/kg (Fig. 3).

**Table 2:** Average residual solid masses of 0.2-mL sample extract before and after automated clean-up with µSPE cartridges (*n* = 3)

	Before µSPE	After µSPE	% residual mass removed
Lettuce ITSP (EtOAc)	0.4 mg	0.4 mg	8%
Raspberry ITSP (EtOAc)	13.5 mg	5.3 mg	61%
Egg ITSP (EtOAc)	7.1 mg	5.1 mg	27%
Avocado ITSP (EtOAc)	18.9 mg	13.9 mg	26%
Avocado ITSP (MeCN)	1.7 mg	0.5 mg	73%
Paprika ITSP (EtOAc)	4.3 mg	2.4 mg	44%
Paprika PAL System (EtOAc)	4.3 mg	2.6 mg	39%
Liver ITSP (EtOAc)	0.4 mg	0.3 mg	33%

In Table S1, all the mean recoveries; the repeatabilities at the spiking levels of 0.001, 0.005, 0.01, 0.05, and 0.1 mg/kg; and the LODs for each matrix and the 212 compounds using the routine ITSP cartridges for clean-up are presented in Excel format as Electronic Supplementary Material. In this large table with 1484 rows, data can be extracted using the filter function. For the paprika spice, it lists the results also for the PAL System cartridges.

Recoveries, relative standard deviations, and LODs are mostly acceptable. The easier the matrix (e.g., raspberry, lettuce), the better the results (Fig. 3) with a higher number of compounds validated and lower LODs < 0.01 mg/kg. For spikes of 0.01 mg/kg upwards, no great changes of the number of validated compounds were noticed.

**Fig. 4:**
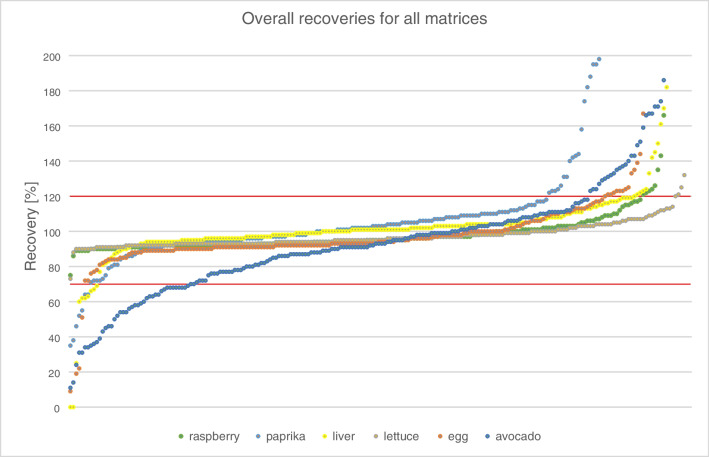
Overall recoveries for all matrices with the ITSP cartridges at 0.1 mg/kg (avocado as an exception extracted with MeCN); the *x*-axis shows the ascending recovery of detected compounds (different number of compounds depending on matrix). The red lines depict the recoveries of 70 and 120%

Figure 4 shows the distribution of recoveries of the substances detectable in the corresponding matrix at 0.1 mg/kg using the routine ITSP cartridges for clean-up. In general, easier matrices such as lettuce and raspberry show a higher number of substances with recoveries between 70 and 120%, while the number of substances outside this range increases for more difficult matrices. The number of compounds with recoveries in the range of 70–120% is 207 of 212 (98%) for lettuce, 195 (92%) for raspberry, 182 (86%) for liver, 177 (83%) for egg, 156 (74%) for paprika, and 142 (67%) for avocado.

In avocados with MeCN extraction, a rather high number of 41 compounds show recoveries of < 70%. Many of these compounds are rather apolar (e.g., hexachlorbenzene and some PCBs). The low recoveries may be due to lower solubility of these substances in MeCN compared to EtOAc and thus a partial partitioning into the avocado lipids during extraction.

No optimization was needed for lettuce, raspberry, liver, and egg (Table S1, Fig. 4). For these four matrices, the validation criteria (e.g., recovery range 70–120% and RSD ≤ 20%) at 0.1 mg/kg were fulfilled in lettuce, raspberry, liver, and egg for 207, 195, 182, and 177 of 212 compounds, respectively (Fig. 3). No chromatographic effects, e.g., retention time shifts or peak deterioration, were observed in these matrices.

#### Paprika

Repeated injections of paprika EtOAc extracts with the ITSP cartridge showed some analyte-specific shifts in retention times between samples and external standards. Nevertheless, 74% (156 compounds) of the analytes passed the validation criteria at 0.1 mg/kg (Fig. 3). Although these retention time shifts complicate processing of the raw data considerably, the recoveries lie in a similar range, compared to the other matrices.

To address the above observed shift effects and to further optimize recoveries, the novel PAL System µSPE cartridges designed by CTC Analytics with a customized sorbent mixture for complex matrices such as tea or spices were also employed. Composition of the routinely used ITSP µSPE cartridges and the PAL System µSPE cartridges is given in Table 1.

The retention time shifts between samples and standards could not be reduced using the complex matrix cartridges by the PAL System. The masses of the residual solids after clean-up with the ITSP and the PAL System cartridges are similar (Table 2), whereas the residues after clean-up with the PAL system cartridges have a significantly lower coloration. This indicates that the clean-up of the paprika extracts is better with the PAL system cartridges than with the ITSP cartridges.

Some compounds that showed good recoveries between 70 and 120% at 0.1 mg/kg after clean-up with ITSP cartridges showed higher recoveries of > 120% after clean-up with the PAL System cartridges, e.g., hexachlorobenzene, imibenconazole, parathion-ethyl, or pyrimethanil. This effect is attributed to losses of these analytes in the external standard (prepared with cucumber matrix as the analyte protectant) which is a much weaker matrix than the paprika samples examined. As a result, active areas in the µSPE cartridges are not entirely covered by the matrix. Other substances do not show any major differences in recovery after cleaning with the two different µSPE cartridges, e.g., PCBs, bifenthrin, cypermethrin, chlorpyrifos, or phosmet. For PAHs, no recoveries could be calculated due to possible losses of these substances when GCB (contained in the PAL System cartridge) is used for clean-up. This effect was also observed by Nicolas Michlig and Steven Lehotay [30]. When using the PAL complex matrix cartridges, 59% (125 compounds) of the analytes passed the validation criteria at 0.1 mg/kg (recoveries within the range 70–120% and RSD ≤ 20%).

### Operational performance

**Fig. 5:**
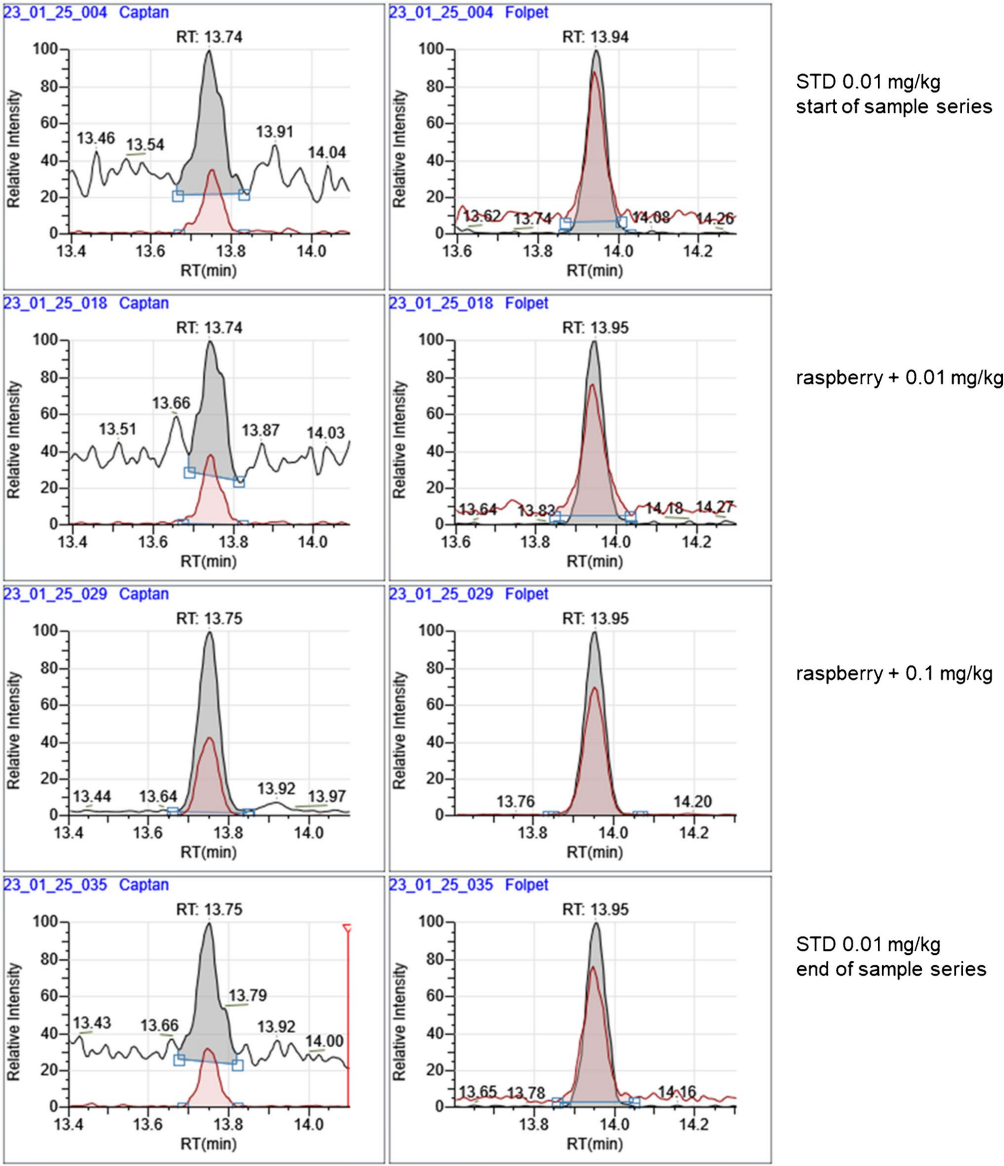
Extracted chromatograms of raspberry samples spiked with captan (left) and folpet (right) after µSPE clean-up

It could be shown that the difficult-to-analyze pesticides folpet and captan can be detected in EtOAc extracts at low levels of 0.01 mg/kg as demonstrated in Fig. 5 for raspberry samples. In paprika and egg, these substances were not detectable even at 0.1 mg/kg. It is also crucial for the analysis of captan and folpet that the GC inlet liner and the analytical column are in good condition. In routine analyses using the µSPE clean-up, a clean inlet liner could be maintained over more than approximately 200 injections, depending on the matrix.

The GC-MS system runs about 100 sample injections per week, in addition to the calibration and system suitability checks. As a result of the applied µSPE raw extract clean-up, a liner exchange is usually performed only once a week, reducing system downtime significantly. Even at the time of change after about 100 sample runs, the liner still appears to be clean without visible residues.

The lower matrix burden after the µSPE raw extract clean-up also shows up with the extended lifetime of the GC column in use. The column gets clipped half a meter only after about 6 months of use and more than 2600 sample analyses run on the system. An MS ion source maintenance is performed, when deemed necessary, approximately once a month. The maximum number of 54 samples in the given configuration of the robot always provided sufficient capacity for overnight sample processing allowing the reporting already the next day.

#### Sequences for validation in contrast to routine

**Fig. 6:**
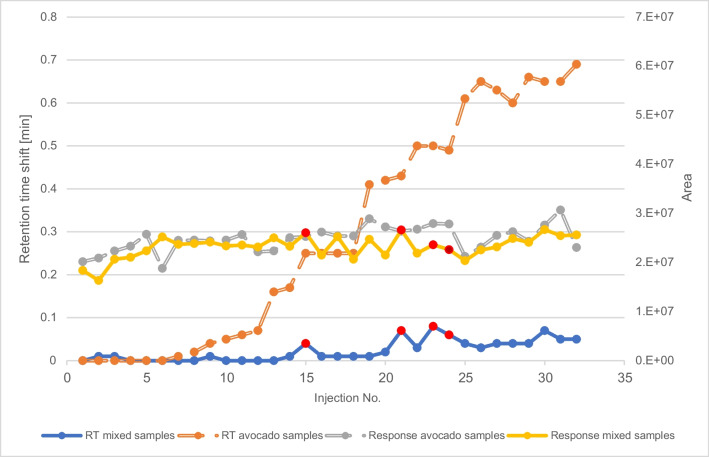
Retention time shift (orange) and response as area of TPP (grey) over a sequence of avocado samples only, in comparison to a sequence of mixed matrices sequence (blue and yellow; both extracted with EtOAc and without freeze-out). The red dots depict the 4 avocado samples in the mixed matrix sequence (basil, cilantro, basil, parsley, spring onion, morning glory, basil, basil, cilantro, orange, avocado, date, date, orange, date, avocado, orange, avocado, avocado, date, pineapple, rice, rice, milk, and pineapple bracketed with 4 standards at the beginning and the end)

One aspect of method validation is the gap between approved method validation strategies involving repeated measurements of the same matrix and everyday practical circumstances encountered in our laboratory. As a small enforcement lab, we hardly ever encounter the situation, where 10 or more samples of the same matrix must be analyzed for residues of pesticides, PCBs or PAHs. Normally, series of 10 to 20 samples consisting of different foods are analyzed. An exemplary series of different matrix samples, including four avocado EtOAc extracts and even other complex matrix extracts such as herbs, is given in Fig. 6, depicting that the deterioration of the method performance is not observed.

Consecutive injections of fatty matrix sample extracts, typical for some matrix-dependent validations, is to be considered as a worst-case scenario, leading to loss of method robustness never observed in real life in our routine analysis. As shown, method validation schemes disqualify many analytes from validation regarding recovery, reproducibility, and retention time due to the stringent method quality parameters while the robustness of the method is not adversely affected, when series of different kinds of samples are run. This demonstrates that the method is fit for purpose in routine operation with such mixed sample series.

#### Quantification

In routine, a screening procedure is used to identify potential non-compliant samples. The residues in the suspected non-compliant samples are quantified in a second step by using the standard addition method with the identified pesticide compensating for possible matrix effects. The interpretation of analytical information depends on compound, expected metabolites, matrix, condition of the liner, column and mass spec, possible carry-over and many other compound, and regulatory specific peculiarities. Finally, the experience of the analyst influences the decision whether to initiate confirmation analyses based on the screening results or not. In our lab, confirmation includes double extraction of the back-up samples, 3-point standard addition, if needed additional dilution, adjustment of sample weight or application of acidic extraction conditions, optimized MS parameters (e.g., more dwell time and mass transitions), and consideration of processing factors, relevant MRLs, and measurement uncertainties.

## Conclusions

The current setup with the ITSP cartridges allows the described automated µSPE clean-up of many matrix types with EtOAc extraction. With this type of µSPE cartridge in routine also samples, such as egg, avocado, or liver, that would have formerly been processed with a customized clean-up, can be run without the need of e.g. a separate freeze-out of fats. Known critical matrices, like spices with a high content of essential oils, and unfamiliar matrices are treated using the described workflow without any alteration. The initial sample weight is adjusted (2, 5, or 10 g) according to suspected matrix properties.

The comparison of the automated µSPE workflow to the earlier manual method using an optimized dispersive (dSPE) clean-up with sorbent mixes for a particular food commodity showed very good compliance within the normal and accepted error range in pesticide analysis.

Another big advantage of automated μSPE coupled to GC-MS/MS for routine analysis is the time saved compared to the previous approach with manual dSPE and dilution, even more so compared with the time-consuming GPC purification for high-lipid matrix containing samples. Also, errors can be avoided by automating the clean-up process of the raw extracts. From our experience, the robot runs reliably without noteworthy crashes or abortion of the workflow. The described µSPE workflow has been in routine operation for 2 years now and showed high reliability also applied for unattended overnight runs, releasing time from earlier manual workload to be used for other duties such as data evaluation and quantitation of the numerous analytes.

For EtOAc extracts of fatty matrix samples such as paprika spice or avocado, the use of one standardized routine μSPE cartridge only, e.g., the ITSP, can be limited, especially when performing consecutive injections. Further optimization of initial sample weight, volume of applied extract in the µSPE, sample dilution or the composition of sorbent mixtures, and amounts in the cartridges is required. Customized cartridges with higher sorbent material volumes and increased capacity can be implemented. The new PAL cartridge design allows bed masses of up to 100 mg.

In routine, it would be easier to use only one type of cartridge for all matrices. The scope of samples using the described workflow will be extended to also include complex matrices (e.g., cheese or composite food) to avoid the time-consuming manual steps such as freeze-out after extraction. Uniform fit-for-routine procedures giving enough method robustness to carry out sample screenings of diverse matrix sequences will be considered.

In conclusion, the combination of EtOAc extraction and automated µSPE with GC-MS/MS represents a promising approach for the sensitive and efficient determination of pesticide residues in diverse food matrices. The proposed method provides a valuable tool for monitoring and controlling pesticide residues in food.

### Supplementary Information

Below is the link to the electronic supplementary material.

Supplementary file1 (XLSX 101 KB)

## Declarations

### Disclaimer

Mentioning of company and brand names does not constitute an endorsement by the Cantonal Laboratory Zürich, Official Food Control Authority of the Canton of Zürich, Department Pesticide Analysis, Zürich, Switzerland, above others of similar nature not mentioned.
